# Cryptosporidiosis after treatment with fingolimod: a case report and pharmacovigilance review

**DOI:** 10.1186/s12879-020-04988-7

**Published:** 2020-03-30

**Authors:** M. Martinot, A. Abou-Bacar, M. Lamothe, M. Alt Tebacher, M. Mohseni Zadeh, F. Dalle, L. Favennec, D. Costa, J. Brunet, F. Sellal

**Affiliations:** 1 Service de Maladies Infectieuses et Tropicales. Hôpitaux Civils de Colmar, 39 avenue de la liberté, 68024 Colmar, France; 2 Laboratoire de Parasitologie et de Mycologie Médicale, Plateau Technique de Microbiologie, Hôpitaux Universitaires de Strasbourg, 1 place de l’Hôpital, 67091 Strasbourg Cedex, France; 3 Centre régional de pharmacovigilance Hôpitaux Universitaires de Strasbourg, 1 place de l’Hôpital, F-67091 Strasbourg, France; 4 Dijon University hospital François Mitterand, Laboratoire de Parasitologie Mycologie, Dijon, France; 5 University of Medicine Pharmacy Rouen EA ESCAPE 7510, Rouen, France; 6 CNR LE Cryptosporidiosis, Santé Publique France, Rouen, France; 7 Institut de Parasitologie et Pathologie Tropicale, EA 7292, Fédération de Médecine Translationnelle, Université de Strasbourg, 3 rue Koeberlé, 67000 Strasbourg, France; 8 Service de Neurologie. Hôpitaux Civils de Colmar, 39 avenue de la liberté, 68024 Colmar, France

**Keywords:** Fingolimod, Cryptosporidiosis, *Cryptosporidium*, Multiple sclerosis

## Abstract

**Background:**

*Cryptosporidium* sp. are common intracellular parasites responsible of severe diarrhea in T-cell-immunocompromised patients. We report the first case of a woman who contracted cryptosporidiosis after treatment with fingolimod, a drug labeled for multiple sclerosis and responsible for marked lymphopenia.

**Case presentation:**

A 60-year-old woman was admitted for abdominal pain diarrhea and fever. The patient suffered from multiple sclerosis and had been treated with fingolimod from august 2017 to september 2018 time of occurrence of the first digestive symptoms. Stool culture was negative but parasitological examination was positive for *Cryptosporidium* sp. Blood biological examination profound lymphopenia of 240/mm^3^ [17 CD4/mm^3^ (7%) and 32 CD8/mm^3^ (14%)]. Fingolimod was stopped, and the patient was put on nitazoxanide 500 mg bid for 7 days. The diarrhea resolved and no relapse was observed. Six other cases were found in the Pharmacovigilance database.

**Conclusion:**

Physicians should be aware of this association and screen for *Cryptosporidium* in cases of diarrhea in patients treated with fingolimod. Patients should be aware of this risk and advise to take appropriate measures to avoid such contamination.

## Background

Fingolimod (Gilenya®) is a sphingosine-1-phosphate receptor modulator, labeled for relapsing/remitting multiple sclerosis, that causes a drastic reduction of lymphocytes in the peripheral blood [1]. Several infections, including bronchitis, nasopharyngitis, central nervous system herpesvirus infections, and more rarely, progressive multifocal leukoencephalopathy, Kaposi sarcoma, CNS toxoplasmosis or cryptococcosis,have been reported in patients treated with fingolimod [1–4]. We report the first case of cryptosporidiosis in a patient treated with fingolimod.

Cryptosporidiosis is caused by *Cryptosporidium* sp., an intracellular protozoan parasite responsible for gastroenteritis in humans and animals worldwide. Human cases are commonly due to two species, *C. hominis* and *C. parvum*. *Cryptosporidium* sp. have a global distribution, and *Cryptosporidium* infections are probably underdiagnosed. Recent data have suggested that the prevalence of stools positive for *Cryptosporidium* sp. may reach 1% in high-income countries and 5–10% in low and middle income countries [5]. The illness is usually asymptomatic or results in mild self-limiting diarrhea in immunocompetent hosts, but it can result in prolonged diarrhea (7–14 days), persistent diarrhea (> 14 days), or even life-threatening episodes in malnourished children or T-cell-immunocompromised patients [5–8]. Cryptosporidiosis has been reported as one of the three most frequent causes of death in children aged < 5 years [9]. In France between 2015 and 2017, 210 cases of cryptosporidiosis were reported in immunodeficient patients, mainly patients undergoing solid organ transplantation or HIV infected patients [8], However no earlier report of cryptosporidiosis has been associated with immunocompromised state due to treatment with fingolimod.

## Case presentation

**Fig. 1:**
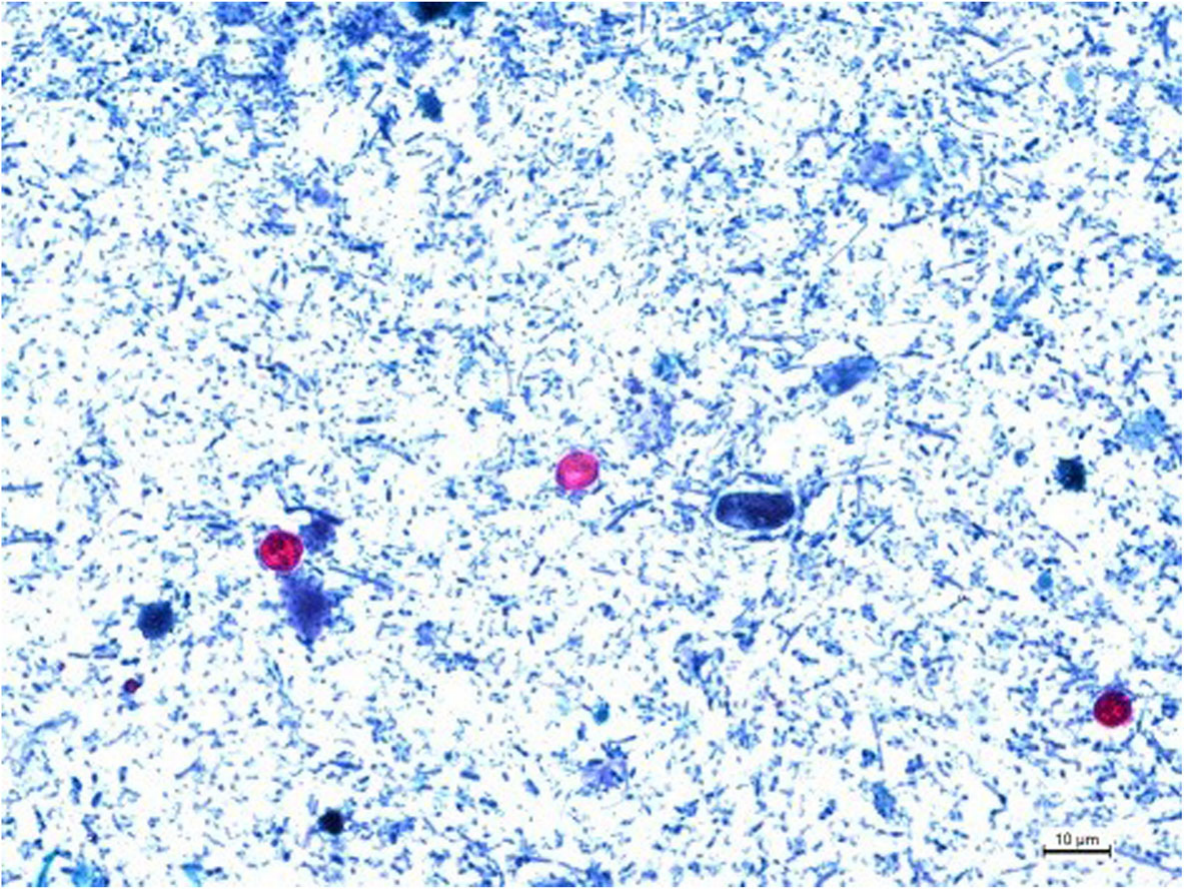
*Cryptosporidium* oocysts from stool, stained with modified Ziehl–Neelsen acid-fast stain. 54x40mm (220 × 220 DPI)

We report the case of a 60-year-old woman who contracted cryptosporidiosis after treatment with fingolimod for multiple sclerosis. The patient was initially treated by interferonβ, and due to underlying pathology progression, fingolimod was introduced in august 2017. The patient was still treated by fingolimod in september 2018 when she presented to our emergency ward for abdominal pain and fever. Blood analysis revealed a mild inflammatory syndrome [C-reactive protein (CRP) level, 75 mg/l (normal range 0-5 mg/l)]. Treatment with amoxicillin and clavulanic acid was initiated. Fever disappeared, but abdominal discomfort persisted, with occurrence of diarrhea 3 days later (5–10 stools/day). Stool culture was negative, including for *Clostridium difficile,* but parasitological examination was positive for *Cryptosporidium* spp. (multiplex gastrointestinal parasite panel PCR Becton-Dickinson BD max®). The patient was referred for an infectious disease consultation. At admission on September 272,018, she still had severe diarrhea with more than seven stools a day and abdominal discomfort. Blood biological examination showed a CRP level that returned to normal (< 5 mg/l) with profound lymphopenia of 240/mm^3^ [17 CD4/mm^3^ (7%) and 32 CD8 /mm^3^ (14%), flow cytometry BD FACS Canto II]. Stool examination using a modified Ziehl–Neelsen staining method revealed the presence of *Cryptosporidium* oocysts (> 10 oocysts/slide, 10 mg stool sample/slide) (Fig. 1). The stool samples were sent to the French *Cryptosporidiosis* national reference center for molecular identification. DNA was extracted from the stool samples with a QIAamp power fecal DNA kit (Qiagen®, Courtaboeuf, France). GP 60 genotyping according to the protocol described by Sulaiman et al. (PCR 1: AL3531 (5′-ATAGTCTCCGCTGTATTC-3′) and AL3533 (5′-GAGATATATCTTGGTGCG-3′); PCR 2: AL3532 (5′-TCCGCTGTATTCTCAGCC-3′) and LX0029 (5′-CGAACCACATTACAAATGAAGT-3′).) revealed IbA10G2 *C. hominis* infection [10]. Fingolimod was stopped, and the patient was put on nitazoxanide 500 mg bid for 7 days [11]. The diarrhea resolved within 4 days, and the lymphopenia rised to 480/mm^3^ lymphocytes [102/mm^3^ CD4 (28%) and 56/mm^3^ CD8 (15%)]. A control parasitological examination of the stool performed 2 weeks later was negative.

## Discussion and conclusion

Fingolimod acts as a functional antagonist of sphingosine-1-phosphate (SP1), a mediator involved in the egress of lymphocytes from lymphoid organs and their recirculation in peripheral compartments, including a subset of lymphocytes implicated in multiple sclerosis [1, 12]. Fingolimod is generally well tolerated, as trafficking of lymphocytes is altered, but not their numbers or functions [1]. The most common adverse effects are cardiovascular events (bradycardia and atrioventricular block) [1–3]. Lymphopenia is part of the mode of action; it quickly occurs after initiation of fingolimod and usually resolves within 45 to 90 days after drug cessation, although long-lasting lymphopenia has been described [1]. Treatment with fingolimod decreased the absolute number of lymphocytes and all subsets of peripheral circulating lymphocytes especially T helpers and B cells and in a lesser extent cytotoxic lymphocytes and NK. Among T cells, naïve and central memory T cells are the most affected [13]. This lymphopenia is usually well tolerated, but severe infections (mostly *Herpesviridae* infections) have been reported [1–4].

*Cryptosporidium* is a protozoan parasite of medical and veterinary importance that causes gastroenteritis in numerous hosts and has a worldwide distribution [5, 6]. *Cryptosporidium* spp. are a common cause of diarrhea in immunocompetent patients, but the severity is typically dependent on parasite load and host factors, ranging from asymptomatic carriage to life-threatening disease [14, 15]. The disease is most severe in T-cell-deficient patients, especially those with AIDS with CD4 count < 50/mm^3^ [7, 8], therefore the marked lymphopenia resulting from fingolimod treatment could be the source for more severe disease. Our patient presented with persistent diarrhea (> 14 days), a course usually encountered in such immunodeficient patients [11].

To the best of our knowledge, no cases of cryptosporidiosis associated with fingolimod have been reported in the published literature. However, we found 6 cases of cryptosporidiosis listed under fingolimod in the database Vigilyse International database (https://www.who-umc.org/vigibase/vigilyze/).. There was 1 man and 5 women (mean age 42 years), among whom cryptosporidiosis occurred after a period of 7 months to 3 years following the introduction of fingolimod; a prolonged hospitalization was noted in 3 cases but no death was reported. Searching for *Cryptosporidium* sp. which is very small is very difficult in stools, and thus practically requires microscopic analysis with specific stains [16]. Consequently, *Cryptosporidium* oocysts are not systematically searched during standard parasitological examination by microscopy. However, detection of *Cryptosporidium* DNA by polymerase chain reaction (PCR) is more and more frequent and also more sensitive that microscopic detection. PCR usually take part of multiplex gastrointestinal parasite panels, which will probably improve the diagnosis of cryptosporidiosis, as in our case [7, 17, 18].

It is difficult to firmly state that fingolimod was responsible for the development of cryptosporidiosis in our patient, as the disease can occur in immunocompetent patients. However, the time course of the diarrhea and the mode of action of fingolimod are in favor of a strong imputability between exposure to fingolimod and the development of cryptosporidiosis. Physicians should be aware of this association and screen for *Cryptosporidium* sp. in cases of diarrhea in patients treated with fingolimod. Patients should also be made aware of this risk and advised to take appropriate measures to avoid such contamination and exposure. Useful measures to help prevent and control cryptosporidiosis in immunocompromised persons can be found on the US CDC website https://www.cdc.gov/parasites/crypto/gen_info/prevent_ic.html (accessed: 10 March 2020).

## Data Availability

The data that support the findings of this study are available from the corresponding author (MM) is available from the corresponding author upon reasonable request.

## Abbreviations

CNS: Central nervous system
CRP: C-reactive protein
